# Pose Estimation of Excavator Manipulator Based on Monocular Vision Marker System

**DOI:** 10.3390/s21134478

**Published:** 2021-06-30

**Authors:** Jiangying Zhao, Yongbiao Hu, Mingrui Tian

**Affiliations:** National Engineering Laboratory for Highway Maintenance Equipment, Chang’an University, Xi’an 710064, China; jyzhao.chd@chd.edu.cn (J.Z.); tianmingrui1020@163.com (M.T.)

**Keywords:** excavator pose estimation, monocular camera, marker, error analysis, computer vision technology

## Abstract

Excavation is one of the broadest activities in the construction industry, often affected by safety and productivity. To address these problems, it is necessary for construction sites to automatically monitor the poses of excavator manipulators in real time. Based on computer vision (CV) technology, an approach, through a monocular camera and marker, was proposed to estimate the pose parameters (including orientation and position) of the excavator manipulator. To simulate the pose estimation process, a measurement system was established with a common camera and marker. Through comprehensive experiments and error analysis, this approach showed that the maximum detectable depth of the system is greater than 11 m, the orientation error is less than 8.5°, and the position error is less than 22 mm. A prototype of the system that proved the feasibility of the proposed method was tested. Furthermore, this study provides an alternative CV technology for monitoring construction machines.

## 1. Introduction

The construction industry has, for a long time, faced issues surrounding low productivity [1] and high hazard ratios. According to the Ministry of Housing and Urban-Rural Development of the People’s Republic of China [2], 8.41% of accidents were caused by construction machines in China’s construction industry (in 2019). Therefore, it is important to monitor machinery for the management of construction production.

To address this challenge, construction automation (CA) [3] technology has been looked at as a means to improve productivity and safety in construction, which could mitigate dependence on skilled workers, enable safer collaborations between construction machinery and surrounding workers, reduce accidents, and improve productivity. CA has been gradually applied in the construction industry, i.e., by monitoring the environment of construction sites and tracking the movement of construction machines.

On construction sites, CA (e.g., sensor technology) could be used to monitor the construction site environment in real time and return detected information to the operator, which would help the operator understand the surrounding environment (e.g., construction workers and workplace) better. In this way, construction could be conducted more safely and efficiently. On the other hand, monitoring the movement of construction machines (i.e., the pose of an excavator, which includes orientation), combined with other technologies (e.g., task decision and motion planning technology, target detection, and tracking technology), could make construction machines perform actions (e.g., send a warning sound or machine emergency braking) independently, based on the surrounding environment.

There are two primary ways of monitoring construction machines: vision-based [4,5] and sensor-based (i.e., not vision-based) methods. Compared to sensor-based methods, vision-based methods (e.g., UWB, GPS, IMU) [6,7,8] have economic advantages (i.e., they are affordable for many construction contractors to implement). In addition, a vision-based method could provide operators with rich information of the construction sites (e.g., interaction between workers and entities, and wide visual range), which could help operators complete construction projects quickly and accurately. As excavators are the most used construction machines, and their poses are constantly changing, they threaten the safety of workers and surrounding entities. Thus, the precise pose of a construction machine could provide reference information for the operator to adjust excavation work in real time. Furthermore, it could potentially be applied in construction management (such as safety monitoring and productivity analysis).

**Figure 1:**
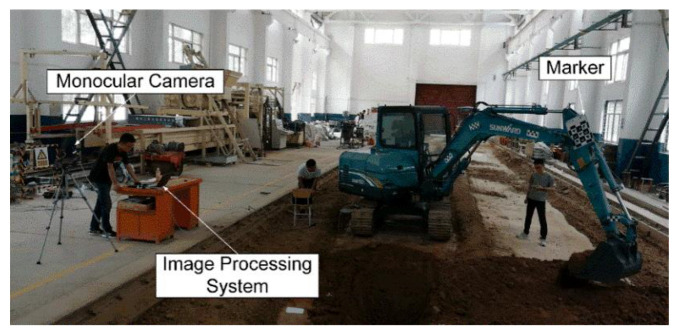
Excavator pose estimation system.

Therefore, it is necessary to monitor the pose of the excavator manipulator in real time, from the perspective of work efficiency and construction safety. In this paper, the approach, based on a monocular vision and marker, was proposed to estimate the excavator manipulator pose. A prototype was established to model the measurement system, which presents sufficient accuracy for implementation through error analysis. As shown in Figure 1, this system mainly consists of a monocular camera, marker, and image processing system.

This work presents the following contributions: first, the authors propose a method of estimating the excavator pose based on a monocular marker system; second, our method could accurately estimate 6D poses rather than a 2D image position, which provides more comprehensive information for construction applications; finally, in this paper, we present a prototype and analyze the accuracy of the pose estimation of this method, showing its feasibility in practical applications.

The remaining parts of this paper are organized as follows: Section 2 reviews work related to the vision-based pose estimation of the excavator; Section 3 presents the pose estimation approach; Section 4 discusses the details of the measurement system design based on the proposed pose estimation approach; Section 5 presents the experimental results and discussion. Finally, the author’s conclusions and future work are summarized in Section 6.

## 2. Related Work

In previous studies, CV technology, such as object detection, object tracking, and pose estimation, was often applied to monitor construction equipment. A complete review of these works is beyond the scope of this paper. Instead, more related studies (e.g., excavator pose estimation) are reviewed in this section. In current practices, there are two types of pose estimation methods used in construction machinery: marker-less and marker-based.

Marker-less methods only require image acquisition equipment and a processing system, which tracks the excavator by extracting image features. Zou and Kim [9] proposed a system to quantify the idle time of hydraulic excavators through the hue, saturation, value (HSV) color space, which could separate excavators from the background. At the same time, the system did not estimate the object’s movement parameters. Rezazadeh Azar and McCabe [10] used a classifier based on histogram of oriented gradient (HOG) detectors to identify excavators with six poses. Then, the server-customer interaction tracker (SCIT) [11] was developed. This system not only identifies the relative interaction between the excavator and truck, but could also measure excavator loading cycles. However, it fails to provide an accurate position and orientation parameters. Yuan et al. [12,13] proposed a binocular vision framework based on a hybrid kinematic shape and key node features to detect, track, and locate the excavators. As for the visual range and depth errors of the binocular vision, it is heavily dependent on a fixed baseline distance. In addition, this system detects key nodes covered by multi-view templates. Thus, the detection system has low robustness, due to the different templates generated at different angles. Soltani et al. [14] presented a method to detect the parts of the excavator manipulator using synthetic images to train the detectors, and then the 2D skeleton of the excavator was extracted. This method requires a synthetic dataset to train the detector and only estimates the 2D orientation [15]. Xu et al. [16,17] described a neural network-based structure to estimate excavator cylinder displacements. Simulation results illustrate that the system could be used to measure the manipulator position, but neural network training requires a lot of data and lacks orientation estimation. Liang et al. [18] proposed a marker-less pose estimation system for 2D and 3D construction robots, which uses the Stacked Hourglass Network [19] of the human pose estimation method. Although this system could detect the robot pose, as well as other methods, it needs to collect a lot of data, and the accuracy of the 3D pose estimation is low [20]. Therefore, further optimization of the algorithm is needed for excavation applications. Torres Calderon et al. [21] used a method of generating excavator pose data through the virtual environment of the Unity 3D engine. The generated dataset is used to train a network model based on deep learning, while analyzing only excavator activity and action prediction. Fang et al. [22] developed a monocular vision technology that combines semantics and prior knowledge to locate architectural entities, using deep learning algorithms to segment the instances in the image, and then foreknowledge models to estimate location information. However, this method requires a large amount of labeled datasets, and position accuracy is limited. Luo et al. [23,24] trained three convolutional neural networks (CNNs) based on the dataset labeled with 2D position information, which was used to estimate the pose of the construction equipment by detecting the key points of the excavator. Subsequently, an recurrent neural network (RNN) named gated recurrent unit (GRU) was proposed, which combined historical data of device movement and activity attributes for pose prediction [25]. However, the above methods need a large amount of data with labeled pose information, which is time consuming and labor intensive. Moreover, as the pose error is relatively large, it is difficult to provide a satisfactory 6D pose.

Marker-based methods detect the marker installed on the excavator manipulator, and the manipulator pose is obtained through CV technology. Compared to marker-less methods, marker-based methods could commonly achieve greater accuracy of pose estimation. Rezazadeh Azar et al. [26] presented a configuration based on a marker-based detecting algorithm to measure the pose of the excavator manipulator [27]. However, this configuration worked with the camera’s optical axis, to be “normal” to the marker plane. Lundeen et al. [28,29] developed an excavator pose measurement system that consists of a marker and an optical system. The system designed four solutions to estimate the final effector pose of the excavator. Therefore, the implementation of the system at construction sites is difficult due to the complexity of the electromechanical system. Feng et al. [30] proposed a structure of camera marker networks. This method shows the feasibility of the system, and the accuracy of the centimeter level measurement was achieved [31]. However, the occlusion resistance was weak, and the orientation error analysis was not considered.

Previous works have shown promising results in excavator monitoring. However, there are still some limitations, such as short visual range or heavy maintenance of binocular cameras and RGB-D cameras. Moreover, few of them focus on accurate monitoring of the overall pose (i.e., position and orientation). Compared to other vision-based methods, the monocular camera is flexible to deploy and simple to maintain on construction sites. Furthermore, the marker helps to increase environment information compared to marker-less methods. Therefore, this work proposes a measurement system that consists of a monocular vision marker system based on the pose estimation method.

## 3. Pose Estimation Approach

**Figure 2:**
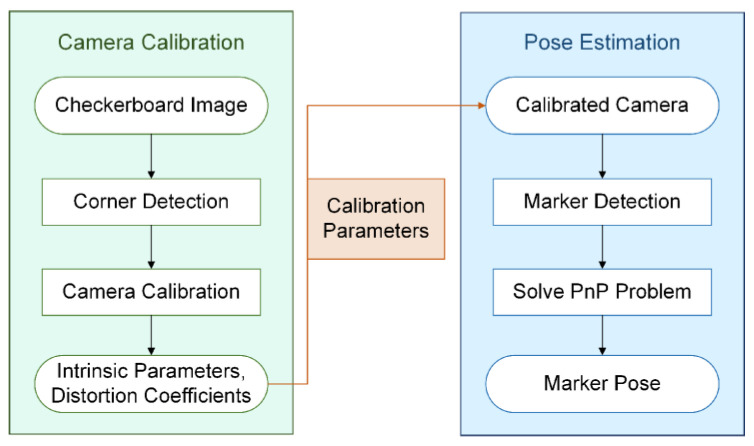
Overview of the pose estimation approach.

Before presenting the structure of the measurement system, the approach of estimating the pose based on the marker is explained. The overview of the approach is shown in Figure 2, which is composed of camera calibration and pose estimation.

### 3.1. Camera Calibration

A camera should be calibrated prior to starting the measurement system to estimate the excavator pose, as shown in the left half of Figure 2. Camera calibration is a critical process to improve system measurement accuracy.

Camera calibration aims to obtain intrinsic parameters, including camera focal lengths in the *x* and *y* directions *f_x_*, *f_y_*, and the main point (the intersection of the image coordinate system plane and the optical axis) (*u*_0_, *v*_0_), and distortion coefficients *k*_1_, *k*_2_, *k*_3_, *p*_1_, *p*_2_. This is achieved in the MATLAB Camera Calibrator Toolbox based on Zhang’s calibration method [32]. It is important to note that, although the pose could also be optimized together with the extrinsic parameters (rotation matrix and translation vector), the optimization solution gets worse with the increase of unknown variables. Therefore, in this paper, intrinsic parameters and distortion coefficients are calibrated before pose estimation.

### 3.2. Pose Estimation

There are several typical marker-based pose estimation methods, including ARTag [33], AprilTag [27], and CALTag [34]. CALTag is a self-identifying marker that could be accurately detected in the image. Compared to other markers, CALTag demonstrated better resistance to occlusion (e.g., CALTag size of 98 × 98 mm had an 88% recognition rate with 25.5% to 32.5% occlusion) [35].

**Figure 3:**
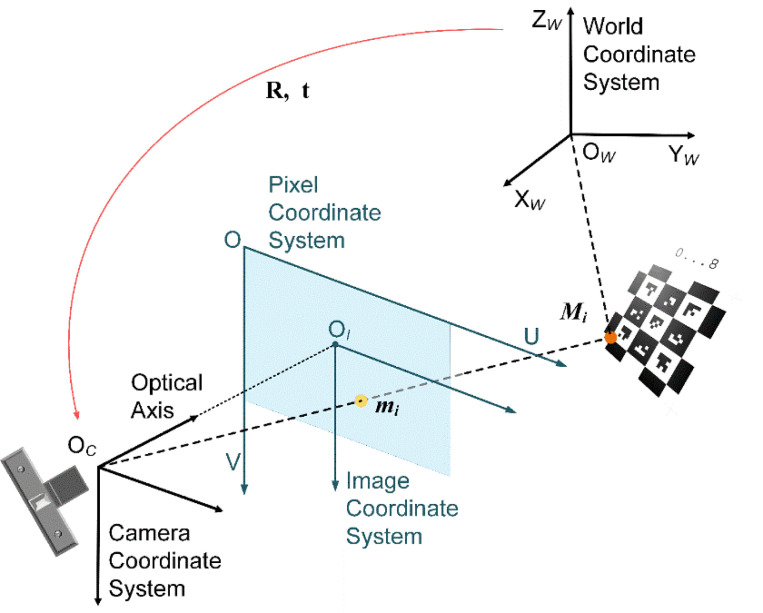
Camera model.

As shown in the right half of Figure 2, the pose is estimated by solving the Perspective-n-Points (PnP) problem [36,37,38,39,40]; that is, estimating the relative orientation and position information between the 3D points in the world and points of the corresponding 2D image. The solution to the PnP problem is based on the camera model (as shown in Figure 3), which is expressed as the following Equation (1):

(1) $$s\left[\begin{array}{c} u\\ v\\ 1 \end{array}\right]=\left[\begin{array}{ccc} f_{x} & 0 & u_{0}\\ 0 & f_{y} & v_{0}\\ 0 & 0 & 1 \end{array}\right]\left[\begin{array}{cc} R & t \end{array}\right]\left[\begin{array}{c} X_{W}\\ Y_{W}\\ Z_{W}\\ 1 \end{array}\right],$$

where ${\left[\begin{array}{cc} u & v \end{array}\right]}^{T}$ are pixel coordinates, ${\left[\begin{array}{ccc} X_{W} & Y_{W} & Z_{W} \end{array}\right]}^{T}$ are world coordinates, *s* is called the scale factor, ***R*** and ***t*** are the rotation matrix and the translation vector, respectively.

Equation (1) can be written as:

(2) $$sp=K\left[\begin{array}{cc} R & t \end{array}\right]P,$$

where ***K*** is called the camera’s intrinsic matrix (usually calibrated before optimization), ***P*** denotes the 3D point, and ***p*** denotes the corresponding 2D point.

The pose is solved by minimizing the re-projection error, which is, minimizing the following function:

(3) $$\underset{R_{i},t_{i}}{\arg\min}\sum_{i=1}^{n}\sum_{j=1}^{m}{\left\|\hat{p}\left(K,R_{i},t_{i},P_{j}\right)-p_{ij}\right\|}^{2},$$

where $\hat{p}\left(K,R_{i},t_{i},P_{j}\right)$ is the projection of the point $P_{j}$ in the *i*-th image.

Solving this nonlinear least squares problem is also called bundle adjustment [40]. The Levenberg–Marquardt algorithm is generally used to solve this problem and the g2o solver [41] could be adopted during the solution of this problem. It should be noted that the EPnP [38] method is used to estimate the camera pose as the initial solution, and then bundle adjustment is used to optimize the estimated value.

### 3.3. Error Analysis

It is inadequate to just estimate pose in a monocular vision marker system. The accuracy of the system needs to be evaluated, which could provide a reference to guide practice. In this paper, the accuracy of the system is evaluated by calculating the absolute error. Given the true camera’s true pose and the corresponding pose estimate value, the absolute orientation and position errors are calculated by the following equations:

(4) $$E_{\mathrm{rot}}=\;\left|r_{\mathrm{true}}^{l}-r^{l}\right|,$$

(5) $$E_{\mathrm{trans}}=\;\left|t_{\mathrm{true}}-t\right|,$$

where $r_{\mathrm{true}}^{l}$ and $r^{l}$ are the ground truth and the estimated value of the rotation matrix in the form of Euler angle (roll–pitch–yaw angle), and *l* takes roll, pitch and yaw angles, respectively. Likewise, $t_{\mathrm{true}}$ and t are the ground truth and the estimated value of the translation vector.

## 4. Design of the Measurement System 

Based on the theory described in the previous section, the measurement system was designed with a monocular camera and marker, and in this section, the details of this system will be presented.

### 4.1. Marker Layout

To obtain the excavator manipulator pose, the marker needs to be installed on the manipulator. First, it needs to determine the size of the CALTag. The size of the CALTag should fit the size of the excavator’s manipulator. It is not convenient to install a larger CALTag on the manipulator. In addition, a smaller CALTag could cause the measurement system to track failure in a long-distance operation. Second, the CALTag should be installed flat on the manipulator, which is useful for the camera to accurately capture the marker. Finally, the marker layout is determined, ensuring that the marker is within the camera’s field of view based on multiple excavation operations.

### 4.2. Monocular Vision Marker System Design

**Figure 4:**
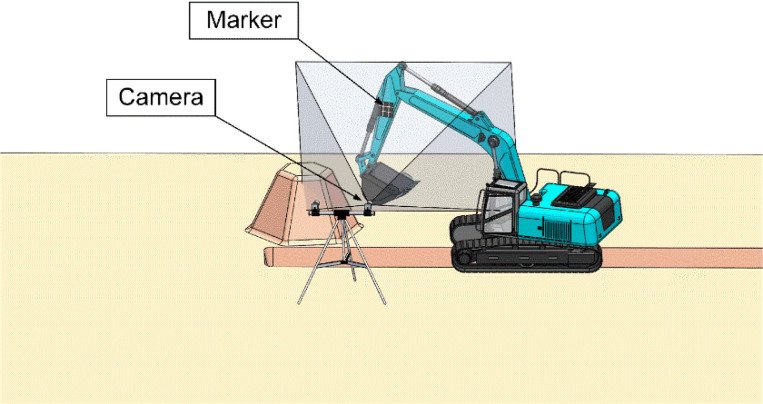
The camera is attached to the side of the excavator.

**Figure 5:**
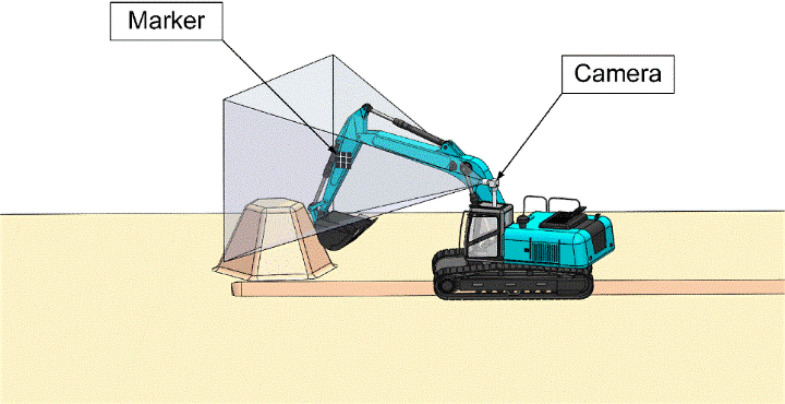
The camera is attached to the excavator cab.

There are two ways to estimate the pose using the camera to capture the marker. One way is to install the camera on a tripod and then attach it to the side of the excavator (Figure 4). The placement of the monocular vision system must ensure that the marker is always in the camera’s field of view during the tracking process of the measurement system. As long as the camera is attached to this system and the marker could be detected in real time, the marker pose could be estimated. The other way is to attach a camera to the excavator cab (Figure 5), to ensure the marker is always within the camera’s field of view, meeting the real-time tracking requirements of the manipulator.

However, in practice, certain operations (e.g., land leveling and long-distance trenching) would cause the marker to be out of the camera’s field of view when the camera was placed on the side of the excavator, and the measurement system could not track the marker, causing the system to crash. For this problem, a possible solution is to install more cameras (i.e., single marker and multiple camera system) taking into account the construction site environment. Figure 4 and Figure 5 only illustrate the simple design of the system. All of this leads to system design.

### 4.3. System Prototype

To verify the feasibility of the monocular vision marker system (Figure 1), the experimental model of the measurement system is established. In this paper: first, the marker is installed on a rotatable plate to simulate the movement of the excavator; second, a camera installed on a tripod is adopted to track the marker in real time; finally, the excavator pose is calculated by the image processing system.

## 5. Results and Discussion

In this section, three sets of experiments were performed and the results were analyzed. In addition, the authors discuss limitations and applicability compared to existing vision-based pose estimation methods.

### 5.1. Results

#### 5.1.1. Effectiveness Experiments of Measurement System 

Before implementing this system to estimate marker pose, a group of experiments was carried out under different conditions to assess the effectiveness of the measurement system.

Considering the size of the excavator manipulator (a 5-ton excavator, with a manipulator size of 200 to 400 mm in width), a CALTag with a size of 228 × 228 mm was adopted as the marker, and the image resolution was 1280 × 960 pixels. It should be noted that the normal lighting condition was considered only in these experiments. As described in the previous section, the camera should be calibrated before estimating the pose. Table 1 lists the calibration results.

**Figure 6:**
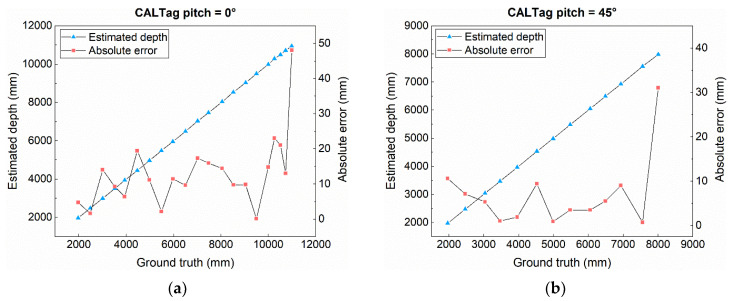
Maximum detectable depth under pitch difference. (**a**) Estimated depth under CALTag pitch = 0°. (**b**) Estimated depth under CALTag pitch = 45°.

Then, the effectiveness of the system was evaluated by calculating the maximum detectable depth. Since distance and angle are the vital factors affecting effectiveness, in this paper, the experiments were performed while the CALTag pitch angle was 0° and 45°, respectively. The experimental results are shown in Figure 6, the maximum detectable depth is greater than 11 m with the 0° pitch (Figure 6a). Furthermore, it could be seen that, when approaching the maximum depth, although the marker could be detected, the absolute error between the ground truth and the estimated depth changes abruptly; this point is considered an outlier (i.e., the maximum detectable depth). Likewise, when the pitch of the marker is 45° (Figure 6b), the maximum detectable depth is about 8 m, which is significantly less than the result of the pitch of the CALTag being 0°.

#### 5.1.2. Orientation Accuracy Experiments

**Figure 7:**
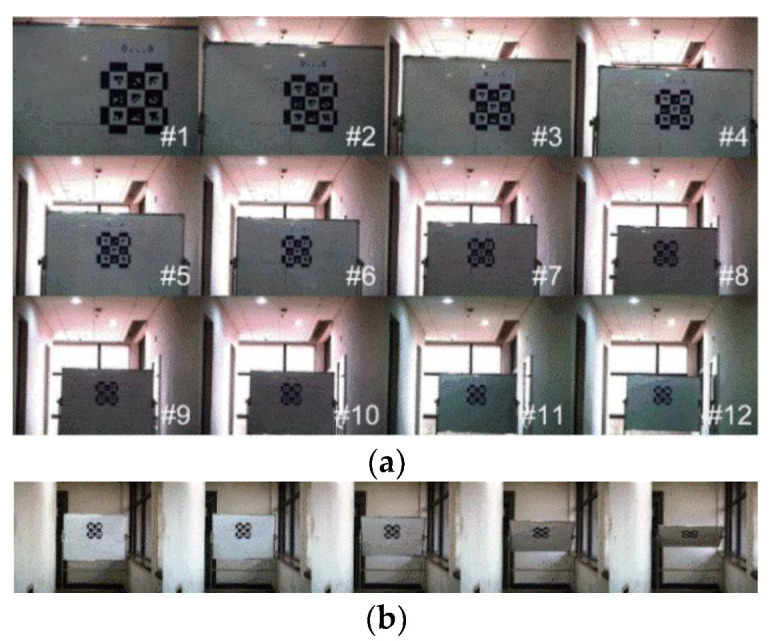
Orientation accuracy experiments with different configurations. (**a**) Different depth. (**b**) Different pitch. # 1 to 12 represents change in depth from 1963 to 7558 mm.

In this paper, we evaluate accuracy by the orientation error that is calculated using Equation (4). When measuring the measurement’s accuracy, the camera was fixed, and mounted at the same height as the bottom-left of the marker. The marker plane was adjusted to be perpendicular to the normal direction of the camera lens, which was free to rotate about a horizontal axis. The orientation angle was measured by the multifunctional angle ruler (with the accuracy of 1/30°) as the ground truth when the pitch angle varied, which compared with the estimated value using the pose estimation approach. These experiments are shown in Figure 7, which shows marker detection under different depth and pitch conditions.

**Figure 8:**
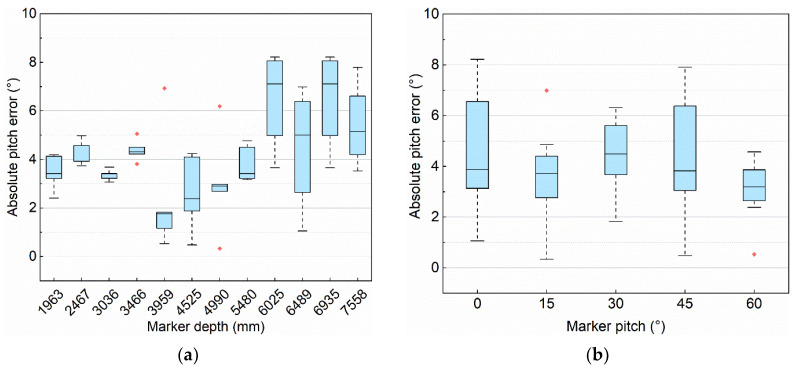
The absolute pitch error with different configurations. (**a**) Absolute pitch error under different marker depths. (**b**) Absolute pitch error under different marker pitches.

Figure 8 shows the absolute error of orientation in different configurations, in these two box plots, the maximum absolute pitch error is less than 8.5° and the average absolute pitch error is about 4° in different configurations.

#### 5.1.3. Position Accuracy Experiments

**Figure 9:**
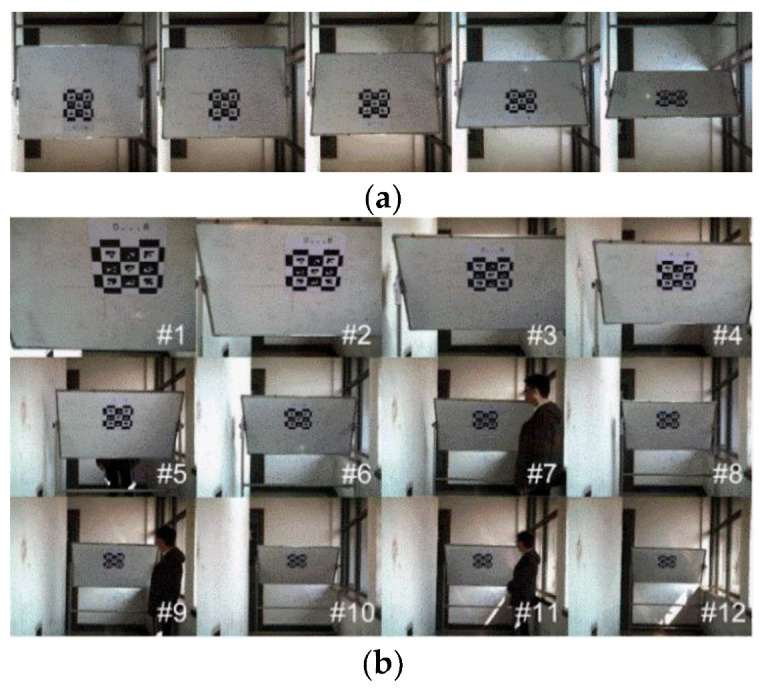
Position accuracy experiments with different configurations. (**a**) Different marker pitch. (**b**) Different marker depth. # 1 to 12 represents change in depth from 1963 to 7558 mm.

In this paper, vertical displacement (i.e., depth) was selected because ground truth measurements could be quickly and accurately obtained using a laser rangefinder, and because depth (*z*-axis) is generally the component of greatest interest in most excavation activities (and a critical factor affecting the method’s feasibility). Position accuracy was completed by calculating the absolute depth error through Equation (5). The estimated value of the measurement system was compared with the ground truth, which was measured by laser rangefinder (with an accuracy of 1 mm). These experiments were tested in different configurations. Figure 9 shows the marker detection under different pitch and depth conditions.

**Figure 10:**
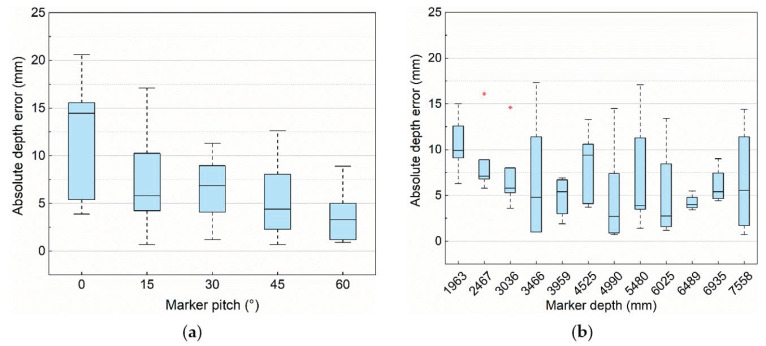
The absolute depth error with different configurations. (**a**) Absolute depth error under different marker pitches. (**b**) Absolute depth error under different marker depths.

As shown in Figure 10, the maximum absolute depth error is less than 22 mm and the average absolute depth error of the pitch angle is about 7 mm. Another prototype experiment (as shown in Figure 1) shows that the absolute maximum depth error is less than 25 mm, which could meet the construction requirements.

### 5.2. Discussion

According to the experimental results, it could be verified that the pose estimation approach is viable in the monitoring of construction machines. While the maximum detectable depth of this work (the error is less than 50 mm when depth is about 11 m) is less than the previous work (the error was 0.5 m when the depth was 15 m), from the results of Yuan’s work [12,13]. This is because marker-based methods allow for better ability to capture key nodes compared to the template-matching method.

The position accuracy of our methodology (maximum error is less than 22 mm) performs similarly to previous studies [30,31] (maximum error was less than 25.4 mm) based on the two cameras. Note that the monocular camera was used, which is convenient for markers placed on a working device with a limited area.

Unlike previous studies [12,13,30,31], orientation accuracy was given in this study. However, it is an unsatisfactory result due to the relatively higher orientation error (the average error is about 4°), a possible reason could be the sensitivity of the rotation matrix. Estimating complete poses (i.e., position and orientation) could provide more comprehensive information for managing production in construction, such as productivity analysis, action recognition, and analysis of the interaction between works and entities. Therefore, it could be concluded that our method achieves a good performance and applicability for estimating the pose of an excavator.

Although the results show that the pose accuracy of the measurement system is sufficient in the construction industry, there are still some limitations that need to be addressed in future work. First, the system’s effective measurement depth is not sufficient in long-distance work (e.g., land levelling, trenching). Thus, it is necessary to increase the working range without increasing the size of the marker. 

A possible solution to overcome this limitation is to model the surrounding environment via LIDAR and register the laser point cloud with the image, which could increase the system’s measurement depth. Second, a large number of markers are inconvenient to install on construction sites, which is time-consuming and labor-intensive. Therefore, excavator characteristics should be fully utilized for detection, such as articulated manipulator activity attributes. 

For this limitation, semantic image segmentation technology could be implemented to track the excavator manipulator, which allows us to separate the manipulator from the background. Then the excavator key points will be extracted, and the pose is calculated using Equation (3). Lastly, similar to other vision-based detection technologies, marker occlusion or insufficient lighting (e.g., night, dense fog) will cause a system failure. More cameras could be installed to prevent occlusion, or other operating methods could be chosen for excavation operations. 

As mentioned in the previous section, this paper intends to explore a form of monitoring technology based on monocular vision, which is flexible for layout under construction. The results of this study provide an alternative solution to the CV technology used in monitoring construction machinery.

## 6. Conclusions

In this work, the authors initially proposed an approach based on monocular vision to automatically estimate the excavator pose by detecting the marker installed on the excavator, which consisted of a marker with significant resistance to occlusion and a common monocular camera. Then, a measurement system was designed to estimate pose based on the pose estimation approach, and an error analysis was proposed to assess the accuracy of the system. Finally, some application guidelines were developed based on the results. The camera was attached to the side of the excavator (a convenient way to view multiple machines with the same camera at construction sites). This approach detected that the system depth was more than 11 m, the orientation error was less than 8.5°, and the position error was less than 22 mm, which could satisfy construction requirements. This prototype and experiments proved the effectiveness of the approach for practical application.

Future work needs to focus on the feasibility of the measurement system in the following aspects: (1) make full use of the excavator’s manipulator structural characteristics and use semantic image segmentation technology to track manipulator movement in real-time, to improve the robustness of the system; (2) expand the working range of the measurement system and improve the versatility of the system; and (3) compare the performance of the measurement system under the different size of the marker. In addition, the fusion of high pixel camera multi-sensor information with inclination sensors could be considered as improving the level of automation of the excavator. Such a fully functional system would likely satisfy the requirements of construction applications.

## Figures and Tables

**Table 1:** Results of the calibration of intrinsic parameters and distortion coefficients.

Calibration Parameters		Calibration Results
Intrinsic parameters (pixels)	(*f_x_*, *f_y_*)	(3172.2, 3144.8)
	(*u*_0_, *v*_0_)	(590.0959, 485.6238)
Distortion coefficients	(*k*_1_, *k*_2_, *k*_3_)	(0.0353, −0.7045, −0.7541)
	(*p*_1_, *p*_2_)	(−0.0015, −0.0043)

## Data Availability

Data sharing not applicable.
